# Tissue-specific pathways and networks underlying sexual dimorphism in non-alcoholic fatty liver disease

**DOI:** 10.1186/s13293-018-0205-7

**Published:** 2018-10-22

**Authors:** Zeyneb Kurt, Rio Barrere-Cain, Jonnby LaGuardia, Margarete Mehrabian, Calvin Pan, Simon T Hui, Frode Norheim, Zhiqiang Zhou, Yehudit Hasin, Aldons J Lusis, Xia Yang

**Affiliations:** 1 Department of Integrative Biology and Physiology, University of California, Los Angeles, Los Angeles, CA USA; 2 Department of Medicine/Division of Cardiology, David Geffen School of Medicine, University of California, Los Angeles, Los Angeles, CA USA

**Keywords:** Non-alcoholic fatty liver disease (NAFLD), Sexual dimorphism, Multi-omics integration, Key regulator genes, Bayesian networks, Coexpression networks, Hybrid mouse diversity panel

## Abstract

**Background:**

Non-alcoholic fatty liver disease (NAFLD) encompasses benign steatosis and more severe conditions such as non-alcoholic steatohepatitis (NASH), cirrhosis, and liver cancer. This chronic liver disease has a poorly understood etiology and demonstrates sexual dimorphisms. We aim to examine the molecular mechanisms underlying sexual dimorphisms in NAFLD pathogenesis through a comprehensive multi-omics study. We integrated genomics (DNA variations), transcriptomics of liver and adipose tissue, and phenotypic data of NAFLD derived from female mice of ~ 100 strains included in the hybrid mouse diversity panel (HMDP) and compared the NAFLD molecular pathways and gene networks between sexes.

**Results:**

We identified both shared and sex-specific biological processes for NAFLD. Adaptive immunity, branched chain amino acid metabolism, oxidative phosphorylation, and cell cycle/apoptosis were shared between sexes. Among the sex-specific pathways were vitamins and cofactors metabolism and ion channel transport for females, and phospholipid, lysophospholipid, and phosphatidylinositol metabolism and insulin signaling for males. Additionally, numerous lipid and insulin-related pathways and inflammatory processes in the adipose and liver tissue appeared to show more prominent association with NAFLD in male HMDP. Using data-driven network modeling, we identified plausible sex-specific and tissue-specific regulatory genes as well as those that are shared between sexes. These key regulators orchestrate the NAFLD pathways in a sex- and tissue-specific manner. Gonadectomy experiments support that sex hormones may partially underlie the sexually dimorphic genes and pathways involved in NAFLD.

**Conclusions:**

Our multi-omics integrative study reveals sex- and tissue-specific genes, processes, and networks underlying sexual dimorphism in NAFLD and may facilitate sex-specific precision medicine.

**Electronic supplementary material:**

The online version of this article (10.1186/s13293-018-0205-7) contains supplementary material, which is available to authorized users.

## Background

Nonalcoholic fatty liver disease (NAFLD) covers a wide spectrum of disorders spanning simple liver steatosis, nonalcoholic steatohepatitis (NASH), cirrhosis, and hepatocellular carcinoma [1–3]. NAFLD has rapidly become a significant health threat globally, affecting 25% of the world population on average, and is strongly associated with insulin resistance, type II diabetes, and obesity [1, 4–6]. Due to the lack of mechanistic understanding of NAFLD, there are no existing therapies directly targeting NAFLD. Moreover, significant unexplained age-dependent sexual dimorphisms have been observed in NAFLD. At younger ages, NAFLD has a higher prevalence in males than females, whereas at older ages, especially after menopause, the prevalence in females increases [7–10]. Males with NAFLD have more severe metabolic phenotypes than females, including higher glucose levels, higher systolic blood pressure, greater visceral adiposity, lower adiponectin levels, lower high-density lipoprotein cholesterol levels, and greater liver injury as measured by alanine aminotransferase levels and aspartate aminotransferase levels [11]. Although endogenous estrogens, adipose distribution, and other lifestyle factors have all been proposed as possible contributors to sex differences in NAFLD [10–13], the molecular mechanisms are unclear. Revealing the underlying biological mechanisms driving the sex differences in NAFLD can enable the identification of novel therapies and preventive strategies to ameliorate the heightening global health threat from NAFLD in a sex-specific and personalized manner.

Recently, human genome-wide association studies (GWAS) have revealed a handful of candidate causal genes such as *PNPLA3*, *SAMM50*, *PARVB*, *GCKR*, *LCP1*, *LYPLAL1*, *PPP1R3B*, *TM6SF2*, and *TRIB1* for NAFLD [14–16]. However, sex differences in genetic risks have not been investigated in these studies. In addition, NAFLD is strongly influenced by environmental factors such as diet, which are difficult to control in human studies. Rodent models, on the other hand, allow for control of environmental factors and collection of molecular traits from the relevant tissues when examining a complex disease. To enable the study of sex-specific mechanisms of NAFLD, hepatic steatosis and its relevant clinical and molecular traits were recently examined in both male and female mice of more than 100 distinct inbred and recombinant inbred strains from the hybrid mouse diversity panel (HMDP) [17]. These mice were treated with a high fat and high sucrose diet to generate hepatic triglyceride accumulation or steatosis, a hallmark of NAFLD. A comprehensive NAFLD-associated and sex-specific multi-omics data resource has also been generated, encompassing genotyping of common genetic variants, transcriptome data from liver and adipose tissue, and tissue-specific expression quantitative trait loci (eQTLs, reflecting the genetic regulation of gene expression in individual tissues). These data sets enable tissue-specific and sex-specific investigations of NAFLD mechanisms.

To fully leverage the multi-omic datasets from HMDP mice and incorporate disease-associated molecular signals with strong, moderate, and subtle effects, we have recently deployed an integrative approach to identify potential causal pathways, gene networks, and key regulators of NAFLD in males in a tissue-specific fashion, followed by experimental validation of the novel predictions [18]. In the current study, we apply this validated approach to compare the NAFLD biology between sexes to uncover the shared, male-specific, and female-specific genes, processes, and networks potentially driving NAFLD pathogenesis, thereby providing more comprehensive tissue-specific insight into the differential prevalence and manifestations of NAFLD between sexes.

## Methods

### Overall study design

**Fig. 1:**
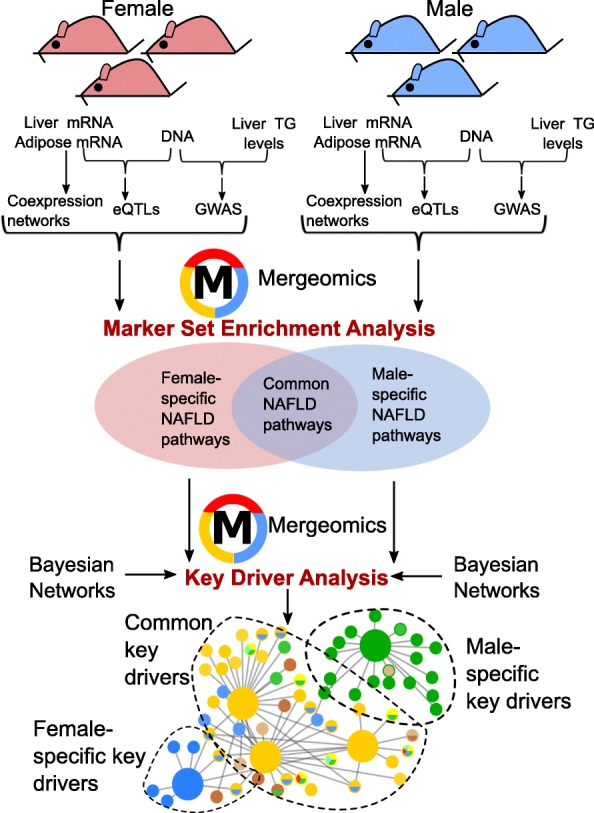
Schematic representation of the methodology. Genotype, liver and adipose tissue gene expression data, and hepatic triglyceride phenotypic data from both sexes of the hybrid mouse diversity panel (HMDP) mice were first integrated using Marker Set Enrichment Analysis in the Mergeomics pipeline to predict sex- and tissue-specific pathways perturbed in NAFLD. Then, potential regulatory genes (key drivers) for male-specific, female-specific, and shared pathways were identified using the Key Driver Analysis in Mergeomics. TG triglyceride, eQTL expression quantitative trait loci, GWAS genome-wide association studies

We aimed to understand the sexually dimorphic mechanisms underlying NAFLD using an integrative genomics approach, Mergeomics [19, 20]. In our recent study [18], we used this pipeline to integrate the multi-omics data from the male mice of the hybrid mouse diversity panel (HMDP) [17, 21] to identify causal NAFLD gene networks and predict key regulator (driver) genes in these networks. Our subsequent in vivo and ex vivo experiments supported the reliability of our multi-omics modeling approach [18]. In our current study, we predicted the NAFLD processes and their key driver genes using the female HMDP mice [17, 21] and compared these findings with those from the male-focused study [18]. As illustrated in Fig. 1, we first reconstructed tissue-specific coexpression networks and identified modules (groups of co-expressed genes) using liver and adipose tissue gene expression data from female mice across more than 100 HMDP strains. Then, we integrated these modules with GWAS of hepatic triglyceride levels together with the eQTLs from liver and adipose tissue to identify gene co-expression modules or biological pathways enriched for NAFLD GWAS signals in females. The incorporation of the genetic signals helped to infer causal modules and pathways that are perturbed by genetic risks. Lastly, these NAFLD-associated gene sets in females were mapped on Bayesian networks that carry gene-gene regulatory information to predict potential key driver genes of the NAFLD processes. The findings from the female-specific analysis were compared with those from the male-specific study [18] to retrieve sex-specific and shared mechanisms.

### HMDP study of NAFLD

The HMDP strains and the NAFLD study design with a high-fat high-sucrose diet were previously described in detail [17, 22]. Experimental procedures had been approved by the UCLA animal research committee. Female mice from 103 mouse strains and male mice from 113 strains used in this study were purchased from the Jackson Laboratory and bred at University of California, Los Angeles. These mice were fed an 8-week chow diet followed by an 8-week high-fat high-sucrose diet with 16.8% kcal protein, 51.4% kcal carbohydrate, and 31.8% kcal fat [17]. They were sacrificed after a 4-h fasting.

### Hepatic lipid content measurement

Liver lipids were extracted from 365 female mice and 465 male mice by following an established method [23]. About 60 mg of liver was used for lipid extraction, and the dried organic extract was dissolved in 1.8% (wt/vol) Triton X-100 [17]. Colorimetric assay from Sigma (St. Louis, MO) (triglyceride, total cholesterol, and unesterified cholesterol) and Wako (Richmond, VA) (phospholipids) were used to determine the amount of liver lipids in each extract according to the manufacturer’s instructions.

### RNA isolation and gene expression analyses of liver and adipose tissues

Flash-frozen liver and epididymal adipose samples from 256 female mice from 103 strains and 288 male mice from 113 mouse strains were weighed and homogenized, as detailed in [17]. RNA was isolated using RNeasy columns and global gene expression was profiled using Affymetrix HT_MG430A arrays for 206 liver and 211 adipose tissues from females and 227 liver and 228 adipose tissues from males (some mice had both liver and adipose tissue samples analyzed, whereas others had only one tissue passing quality control) [22]. ComBat provided in the SVA tool [24] was used to remove batch effects from the gene expression data.

### Statistical analysis

#### Genome-wide association analysis of liver triglyceride and tissue-specific eQTL analysis in females

Genotypes for 365 samples from 103 strains of female mice were determined using the Mouse Diversity Array [25]. SNPs with poor quality, a minor allele frequency of < 5% or a missing genotype rate of > 10%, were removed as previously described [17], resulting in about 200,000 SNPs. Genome-wide association mapping of the liver triglyceride content and tissue-specific eQTLs were previously generated using Factored Spectrally Transformed Linear Mixed Models [26, 27]. We used cis-eQTLs that were defined within ±1 Mb region of the transcription start and end sites of the genes. False discovery rate (FDR) estimated by the *q* value approach [28] was used for correcting for multiple testing. In the adipose tissue, 216,296 *cis*-eQTL associations were used (76,451 unique *cis*-eSNPs and 1954 *cis*-genes), and in the liver, 241,463 *cis*-eQTL associations were used (81,076 unique *cis*-eSNPs and 2168 *cis*-genes) at *P* < 1E−6 (FDR < 0.01).

#### Reconstruction of tissue-specific co-expression networks from liver and adipose tissue transcriptome data from females

Tissue-specific coexpression networks from gene expression data from 206 liver and 211 adipose tissue of female mice in HMDP were constructed using two complementary network methods: Weighted Gene Co-expression Network Analysis (WGCNA) [29] and Multiscale Embedded Gene Co-expression Network Analysis (MEGENA) [30]. WGCNA tends to cluster genes into large-sized modules and assigns each gene into a single module, whereas MEGENA defines smaller, more coherent modules and can assign each gene into multiple modules. As shown in our recent study focusing on male NAFLD [18], these two methods complement each other and can uncover hidden biology that could be missed by the other method.

Both network methods assign the co-regulated genes into the same coexpression module via hierarchical clustering based on correlation of gene pairs. WGCNA is based on agglomerative hierarchical clustering, whereas MEGENA uses a divisive method of clustering. Gene clusters are merged (in agglomerative) or split (in divisive) according to a distance measure. The distance measure used in WGCNA is Topological Overlap Matrix (TOM) subtracted from 1 (dissTOM = 1-TOM), which is based on the edge weight (correlation score) between two nodes (genes) by considering the edge weights of their neighbors in the network. Distance between two clusters is calculated as the average dissTOM score of all the gene pairs (one gene from each cluster in a pair-wise manner). Divisive clustering of MEGENA is based on the shortest path distance measure and a nested *k*-medoids clustering that finds *k* optimal clusters at each step by minimizing the shortest path distance within each cluster to define more compact modules. The nested clustering process continues until there is no more compact child cluster found. MEGENA clusters genes in a multi-scale manner, allowing the assignment of a gene into multiple modules, but at different scales.

For each module from WGCNA or MEGENA, we annotated the putative biological functions using known biological pathways curated from MSigDB database [31], which incorporates KEGG, Reactome, and Biocarta pathways, using the one-tailed Fisher’s exact test. Bonferroni correction was used to correct the *P* values. Pathways that have an adjusted *P* < 0.05 and shared ≥ 5 genes with a given module were deemed significant. Up to top five significant pathways were used to annotate each module. For modules without significant annotation terms, we used a less stringent cutoff at uncorrected *P* < 5E−3 and ≥ 5 shared genes to annotate them with suggestive pathways (indicated with an asterisk sign in figures and tables).

#### Module preservation analysis of the sex- and tissue-specific coexpression modules

We evaluated the preservation of the female-specific coexpression modules within the male-specific modules and vice versa, in a tissue-specific manner, using the modulePreservation procedure from the WGCNA R package. This procedure reports a *Z*-summary score to determine whether a module is preserved in another data set by using both connectivity and density statistics of the nodes within a module. A *Z*-summary score > 2 means that there is evidence for the preservation of a module in the second condition/dataset tested [29]. The modulePreservation procedure was applied in a tissue-matched manner between sexes to analyze the reciprocal preservation of the modules obtained from the female mice expression data with those from the male mice.

#### Filtering the coexpression modules based on their correlation with NAFLD

For downstream analysis, we only kept the coexpression modules that are relevant to the phenotype of interest (liver triglyceride levels), based on Pearson correlation between liver triglyceride and eigen genes of MEGENA and WGCNA modules. To select NAFLD-correlated modules, correlation *P* < 1E−3 was chosen as a cutoff, which corresponds to a false positive rate of 0.1 based on a permutation test. We generated 1000 random gene sets, with a member size ranging from 20 to 500 genes, as our negative controls, followed by calculating the Pearson correlation between the trait and the eigen gene of each negative control gene set. Among the 1000 negative controls, 102 gene sets had a correlation *P* < 1E−3 with the trait, representing false positives. The remaining negative control gene sets were true negatives. Therefore, at *P* < 1E−3, *false positive rate* = *false positives*/(*false positives* + *true negatives*) = 0.1.

#### Mergeomics pipeline for multi-omics integration

Similar to our previous study on male NAFLD [18], in the current study, we integrated the genetic (liver triglyceride GWAS) and functional genomics data (eQTLs, coexpression modules, and pathways) from female HMDP to define pathways and coexpression networks that are genetically associated with NAFLD using the Marker Set Enrichment Analysis (MSEA) in Mergeomics [19, 20]. MSEA maps the genes within each pathway (from Biocarta, KEGG, and Reactome) or coexpression modules (from MEGENA and WGCNA) to the expression single nucleotide polymorphisms (eSNPs) through eQTLs of the corresponding tissue in female mice. The *cis*-eQTLs (within ±1 Mb of the transcription start and end sites) at *P* < 1E−6 were used for mapping and eSNPs in linkage disequilibrium were trimmed to keep only one eSNP per linkage disequilibrium block based on the block information reported by the PLINK2 tool [32]. The mapped eSNPs for each module or pathway were queried for the corresponding association *P* values from the hepatic triglyceride GWAS. Then, a modified chi-square statistics, which summarizes enrichment assessment across a range of quantile-based cutoffs for the GWAS, is applied to each eSNP set to assess the significance of enrichment for stronger disease-associated GWAS *P* values by comparing the GWAS *P* values of the given eSNP set against the eSNPs of randomly generated gene sets [19]. Since this approach is not based on a single GWAS *P* value cutoff but uses a set of quantile-based cutoffs, it produces more stable enrichment scores and avoids artifacts. This modified chi-square statistics is defined as $\chi ={\sum}_{i=1}^n\frac{O_i-{E}_i}{\sqrt{E_i}+\kappa }$, where *O* and *E* are the observed and estimated number of positive findings (i.e., findings above the *i*-th quantile point), respectively; *n* is the number of quantile points (10 quantile points were used ranging between the top 50% and top 99.9% signals based on the rank of the GWAS *P* values), and *κ* = 1 denotes a stability parameter that reduces the artifacts for small eSNP sets with low expected counts. Benjamini-Hochberg approach was used to correct for multiple hypothesis testing and an FDR < 0.05 cut-off was used to define the significantly enriched gene sets for a given GWAS.

If the significant gene sets (pathways or modules) for NAFLD at FDR < 5% had significant sharing of member genes, defined as gene overlapping ratio > 0.33 and Bonferroni corrected Fisher’s exact test *P* value< 0.05, we merged the overlapping gene sets into non-overlapping “supersets” to reduce the redundancy. Occasionally, we observed that a canonical pathway and a coexpression module, or two independent coexpression modules, which were annotated with the same biological term but did not share significant numbers of genes. In such cases, they were kept as independent supersets despite being annotated with the same (or similar) terms.

To predict potential key regulators, termed key drivers, within the NAFLD supersets, the Weighted Key Driver Analysis (wKDA) from Mergeomics pipeline was used. wKDA projects the tissue-specific NAFLD-associated gene sets onto a tissue-specific Bayesian network, which depicts putatively causal relationships between genes, to identify network hub genes (i.e., key driver genes), whose network neighborhoods are significantly enriched for genes in the NAFLD-associated supersets compared to the neighborhood of a random gene in the network. This analysis has been previously shown to successfully derive meaningful biological findings [19, 33, 34]. Here, we mapped the hepatic triglyceride-associated supersets onto previously defined liver and adipose tissue Bayesian networks, which were curated from multiple human and rodent expression datasets of previous studies [35–41] and constructed using RIMBANET [42, 43] based on the gene expression patterns, genetic information, causal inference, and previously known regulatory relationships among the genes [18]. Since the Bayesian networks incorporate genetic data, they can reveal causal regulatory relationships between gene pairs and enable the identification of potential regulators of disease genes and pathways. While combining the Bayesian networks from these individual studies and defining a union network for each tissue, we did not consider the edge weights and the directions since some of the edge directions included in these Bayesian networks might be conflicting while the edges included in each individual network were considered robust. Gene symbols given in the network figures are illustrated in human orthologs since the curated networks were taken from both human and rodent studies. Key drivers of a NAFLD superset were identified based on a modified chi-square statistics, as described for the MSEA above, that evaluates the enrichment of the member genes in the superset within the candidate key driver’s neighborhood in the Bayesian network compared to that of a random gene chosen from the same network. Benjamini-Hochberg FDR was calculated, and genes with an FDR < 0.05 were determined as significant key drivers of a given NAFLD superset. We identified the top key drivers in each shared and sex-specific NAFLD-superset based on their wKDA-FDR scores. Then, we extracted the subnetworks of the top key drivers in each Bayesian network by gathering the network neighbors of these key drivers.

#### Assessing overlap between the sex-specific NAFLD networks and sex-hormone target genes

To explore the origin of the sex-specific NAFLD networks, we investigated whether the female networks are enriched for estrogen target genes and whether the male networks are enriched for androgen target genes. Liver and adipose transcription factor regulatory networks were obtained from the Functional Annotation of the Mammalian genome (FANTOM) repository [44]. Tissue-specific downstream genes of estrogen receptors and androgen receptors were extracted from the FANTOM networks and used to assess overlaps with the female and male NAFLD networks using Fisher’s exact test in a sex and tissue matched manner (e.g., female liver NAFLD network genes were overlapped with estrogen receptor target genes in the FANTOM liver network).

#### Curation of previously studied NAFLD-relevant genes

As described in [18], 107 previously validated NAFLD-associated genes were taken from the DisGeNET database [45], which manually curates the gene-disease associations via text mining or from databases such as UniProt, ClinVar, Comparative Toxicogenomics Database (CTD), and the GWAS Catalog. We compared these genes to the ones identified by the current study as an in silico validation of our findings.

#### Gonadectomy and ovariectomy

The gonadectomy and ovariectomy experiments were performed as detailed previously [21, 46]. Male and female C57BL/6 J mice were purchased from The Jackson Laboratory (Bar Harbor). Mice were maintained on a chow diet (Ralston Purina Company) till 8 weeks of age and then placed on a high-fat high-sucrose diet (Research Diets D12266B) until 16 weeks of age. At 6 weeks of age, the mice were gonadectomized under isoflurane anesthesia (*n* = 4/group). Scrotal regions of male mice were bilaterally incised, testes removed, and the incisions closed with wound clips. Ovaries of female mice were removed through an incision just below the rib cage. The muscle layer was sutured, and the incision closed with wound clips. In sham-operated control mice, incisions were made and closed as described above. The gonads were briefly manipulated but remained intact.

#### RNA library preparation and sequencing for liver and adipose tissues from gonadectomized or ovariectomized mice

Upon sacrifice, liver and gonadal adipose tissues were instantly frozen in liquid nitrogen. Frozen tissues were homogenized in Qiazol, and following chloroform phase separation RNA was prepared from the pink phase using Qiagen miRNAeasy kits as per original protocol. BioANAlyzer was used to validate total RNA quality (all samples had RIN > 8). RNA libraries were prepared by the sequencing facility at the UCLA Neurosciences Genomics Core using Illumina TruSeq Stranded kits v2, followed by paired-end sequencing. Reads were aligned using STAR 2.5.2b, mm10 genome, and GENCOD M11 transcript annotation. Reads-per-gene tables were generated as part of STAR output, and DESeq2 was used for differential expression analysis (see below).

#### Differentially expressed gene analysis between gonadectomized/ovariectomized mice and sham-operated mice

DESeq2 R Bioconductor package [47] was used to identify the genes that were differentially expressed between the gonadectomized or ovariectomized mice and sham-operated mice. DESeq2 tool estimates the variance-mean dependence of the raw gene counts using a negative binomial distribution and identifies the differentially expressed genes between two groups (i.e., calculates the log2 fold changes and corresponding *P* values for each gene) based on a generalized linear modeling. Benjamini-Hochberg adjusted *P* < 0.05 was used to identify the differentially expressed genes. Then, we analyzed enrichment of the differentially expressed genes for the sex-specific NAFLD subnetworks in the liver and adipose tissues using Fisher’s exact test.

## Results

### Tissue-specific coexpression networks in females

To retrieve functional gene-gene relationships, gene coexpression networks were constructed based on transcriptome data of 206 liver and 211 adipose tissue samples of female mice from 103 HMDP strains using WGCNA [29] and MEGENA [30] (see the “Methods” section). For females, we identified 30 coexpression modules in the liver and 30 modules in adipose tissue using WGCNA, whereas with MEGENA, we identified 213 and 85 modules in the liver and adipose tissue, respectively. These findings are comparable with those from male mice [18]. Comparison of the modules between females and males revealed high preservation, with ~ 95% and ~ 90% of the modules reciprocally preserved between sexes in the liver and adipose tissues, respectively (Additional file 1). However, it is possible that different modules may be perturbed in NAFLD in each sex by different genetic risk factors.

### Identifying modules that are correlated with NAFLD phenotypes in females

Coexpression modules that are associated with the NAFLD phenotype were identified based on the correlation between the expression patterns of the module eigengenes and hepatic triglyceride levels across all female samples (see the “Methods” section). We found 65 MEGENA modules (29 from liver and 36 from adipose) and 10 WGCNA modules (7 from liver and 3 from adipose) to be significantly correlated with hepatic triglycerides. In the male HMDP data, we found similar percentages of adipose modules to be associated with NAFLD [18], but a higher percentage of NAFLD-associated liver modules (32% vs 13% for MEGENA modules and 35% vs 23% for WGCNA modules in males vs females). These percentages suggest less prominent NAFLD-related liver gene network perturbations in females compared to males, agreeing with the milder measures of liver injury in NAFLD females [11]. Functional annotation of the NAFLD-associated modules in females revealed diverse pathways ranging from various metabolic pathways, immune pathways, to extracellular matrix organization (Additional file 2). These NAFLD-correlated modules were further integrated with GWAS signals from female HMDP to identify potential causal mechanisms, as detailed below.

### Biological pathways and coexpression modules that exhibit genetic association with NAFLD

**Fig. 2:**
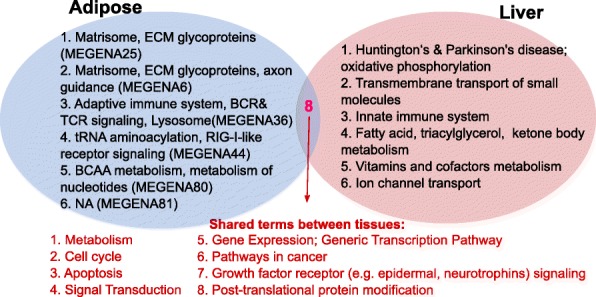
Comparison between NAFLD processes perturbed in the liver and adipose tissue for females. Putative causal pathways that are common to both tissues and unique to each tissue are listed. Co-expression modules are annotated with the most over-represented gene ontology terms. “NA” indicates no over-represented terms were found for a given module. BCAA branched-chain amino acid, BCR B cell receptor, ECM extracellular matrix, TCR T cell receptor

To identify potential causal processes for NAFLD in female mice, genetic information (GWAS; which carries potential causal inference), tissue-specific eQTLs, NAFLD-correlated co-expression modules, and biochemical and signaling pathways were integrated to infer groups of functionally connected genes that together demonstrate significant genetic association with NAFLD in females. Out of the 1823 canonical pathways and the 75 hepatic triglyceride-correlated female modules (36 from liver and 39 from adipose tissue), NAFLD GWAS signals from females were significantly enriched in 18 pathways and 5 liver co-expression modules informed by liver eQTLs, and 12 pathways and 6 adipose modules informed by adipose eQTLs, at FDR < 5% (Additional file 3). Some of the significant modules or pathways share member genes and represent similar biological processes. To reduce the redundancy, we derived “supersets” by merging overlapping gene sets; thereby each superset was comprised of one or more overlapping NAFLD-associated gene sets (see the “Methods” section). These supersets still carry enrichment for strong NAFLD genetic signals that their constituents carried (Additional file 4). Some of the gene sets were annotated with the same term but were not merged because the overlap in gene members was not sufficient to meet our criteria as specified in the “Methods” section (e.g., in female adipose tissue, 2 modules were annotated with extracellular matrix and matrisome terms but were not merged). Comparison of supersets between tissues revealed 6 liver-specific supersets (e.g., innate immune system, oxidative phosphorylation, metabolism of vitamins and cofactors), 6 adipose-specific supersets (e.g., branched-chain amino acid metabolism, extracellular matrix glycoproteins, axon guidance), and 8 cross-tissue supersets (e.g., apoptosis/cell cycle, gene expression regulation, growth factor receptor signaling) (Fig. 2).

### Comparison of the NAFLD-associated supersets between female and male mice

**Fig. 3:**
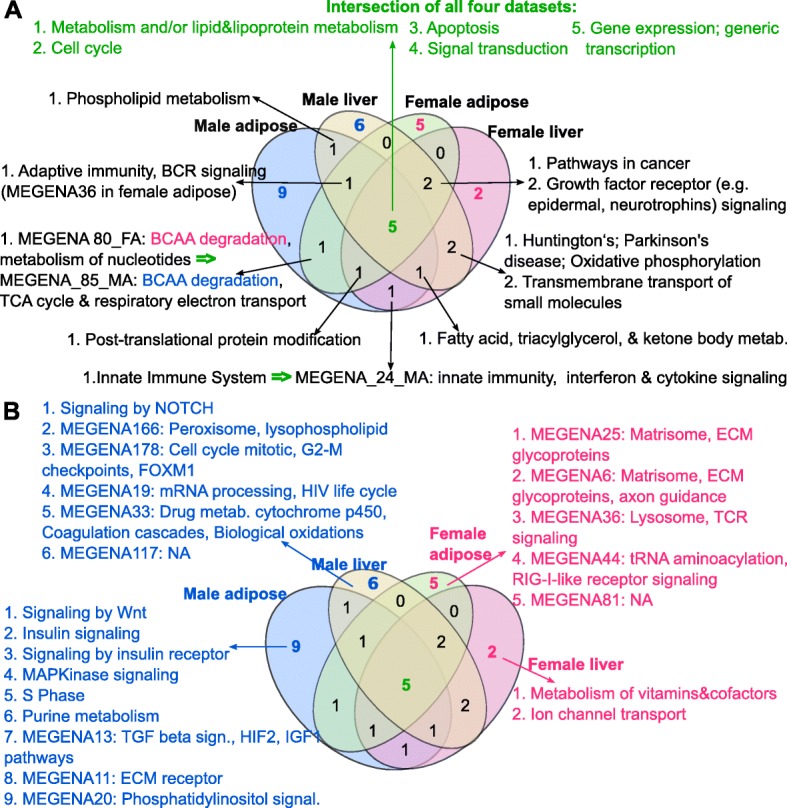
Comparison between NAFLD processes perturbed in the liver and adipose tissue of both sexes. Putative causal pathways that are **a** shared between sexes in one or both tissues and **b** unique to each sex and each tissue are listed. TCA the citric acid

Comparison of the female NAFLD supersets with those from the males [18] revealed five supersets (lipid metabolism, apoptosis/cell cycle, signal transduction, and transcription pathways) to be shared across tissues for both sexes (Fig. 3a; Additional file 4). In liver, oxidative phosphorylation and transmembrane transport of small molecules were shared between sexes, whereas branched-chain amino acid metabolism was shared between sexes in the adipose tissue (Fig. 3a).

Besides the consistent pathways between tissues and sexes discussed above, we also found that certain pathways demonstrate differential tissue-specificity between males and females (Fig. 3a; Additional file 4); innate immunity was specific to liver in females but was specific to adipose tissue in males, whereas the adaptive immune system and B cell receptor (BCR) signaling superset was shared by both tissues in males but was adipose-specific in females. Therefore, the two arms of the immune system appear to act in different tissues during NAFLD in females, whereas both the innate and adaptive immune systems were found to be perturbed in adipose tissue in males. Although the adaptive immune system is implicated in both female and male adipose tissue, it still has a sex-specific pattern since males have transforming growth factor-beta (TGFβ) and BCR signaling pathways, while females have a T cell receptor (TCR) pathway. TGFβ is a cytokine that can induce profibrogenic gene expression and may promote the progression from steatosis to steatohepatitis in males [48]. Additionally, males showed perturbations in fatty acid, triacylglycerol, and ketone body metabolism in both tissues, but this pathway was liver-specific in females. On the other hand, growth factor receptor signaling and cancer pathways were adipose-specific in males but identified in both tissues in females.

Processes that were specific to one sex were also identified. For instance, insulin signaling-related terms were observed only in the adipose tissue of male mice. In addition, more metabolism-related pathways (e.g., lipid, lysophospholipid, fatty acid) were identified in males compared to their female counterparts. Another male-specific adipose pathway was Wnt signaling, which has diverse effects on cellular metabolism and inflammation. Perturbed Wnt signaling is known to contribute to increased inflammation and decreased adipogenesis, leading to triglyceride accumulation and acceleration of NAFLD. Wnt signaling is known to crosstalk with Notch and TGFβ signaling, which were also found to be perturbed only in male liver and adipose tissues, respectively. Peroxisome, cytochrome p450 drug metabolism, and G2-M DNA damage checkpoint are additional male-specific pathways in the liver (Additional file 4). These findings may help explain the more severe metabolic syndrome and liver injury, as well as the higher ratio of DNA damage and inflammation markers observed in males.

For females, metabolism of vitamins and cofactors and ion channel transport were uniquely identified in liver (Fig. 3b), whereas extracellular matrix glycoproteins, axon guidance, and TCR signaling were female-specific in adipose tissue. Ion transport has been associated with oxidative stress and plays an important role in the progression of liver steatosis to insulin insensitivity and more severe conditions such as hepatocellular carcinoma [46, 49–52]. Regarding the vitamins and cofactors pathway, adverse impacts of vitamin A, B12, D, and E deficiency in NAFLD, fibrosis, and NASH have been studied [53–57]. Vitamin A and D are involved in extracellular matrix remodeling during liver fibrosis, B12 deficiency impairs fatty acid oxidation [54], and vitamin E reduces endoplasmic reticulum stress and prevents liver inflammation and apoptosis. Some of these terms have been associated with NAFLD before, but here, we provide novel evidence that they may play a causal role in NAFLD based on the fact that we incorporated genetic signals in our analysis.

### Predicting key driver genes of the NAFLD-associated gene supersets in females

To predict the potential regulators of the NAFLD genes and pathways, we used liver and adipose tissue-specific Bayesian networks [42, 43] that involve causal or regulatory relationships between gene pairs and were independently constructed from previous human and mouse studies [35–41]. Numerous key drivers were predicted for each of the female NAFLD-associated superset at FDR < 5% (top key drivers for each superset in Additional file 4; full list in Additional file 5). We found that 23 of all significant key drivers were among the 107 previously reported NAFLD genes curated in DisGeNET (Additional file 6), including *AHSG*, *FASN*, *RBP4*, and *SREBF1*, which were found to be key drivers in both liver and adipose tissue in our analysis.

**Fig. 4:**
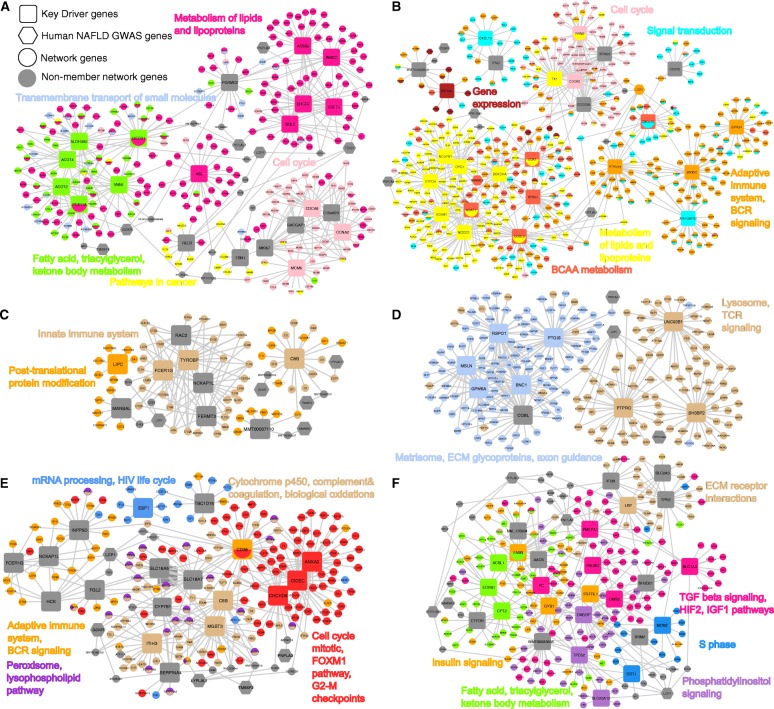
Bayesian gene network representations of NAFLD pathways and their key driver genes. **a** Liver Bayesian subnetwork comprised of shared liver NAFLD supersets between sexes and their top key drivers. **b** Adipose tissue Bayesian subnetwork comprised of shared adipose NAFLD supersets between sexes and their top key drivers. Female-specific **c** liver Bayesian subnetworks and **d** adipose tissue Bayesian subnetworks and their corresponding top key drivers. Male-specific **e** liver Bayesian subnetworks and **f** adipose tissue Bayesian subnetworks and their corresponding top key drivers. Key driver genes are shown with larger node sizes, human GWAS candidate genes are represented in hexagon shapes, and the rest of the genes are represented by smaller node sizes. Each NAFLD-associated superset is indicated with a distinct color in each network. Network genes that are not members of the NAFLD supersets are represented in gray. See also Additional file 4

We compared the top key drivers predicted for the female NAFLD subnetworks with those predicted for males in a tissue-specific manner. As elucidated in Fig. 4 and detailed in Additional file 4, the shared liver or adipose key drivers between sexes included genes with diverse functions involved in fatty acid and cholesterol metabolism (such as *ACOT2*, *DECR1*, *DHCR7*, *SQLE*, *INSIG1*, and *ACSS2*), branched-chain amino acid catabolism (such as *BCKDHA*, *MCCC1,* and *ECHS1*), cell cycle (such as *CDCA8*, *MKI67*, and *CCNA2*), extracellular matrix (such as *FBN1*, *COL1A2*, and *CCDC80*), and immune system and inflammation (such as *RELB*, *IFNG*, and *CXCL10*). The key drivers and the pathways they regulate intimately interconnect in gene networks (Fig. 4a for liver and Fig. 4b for adipose tissue).

We also identified key drivers (Additional file 4) and their associated subnetworks (Fig. 4c–f) for the sex-specific mechanisms in a tissue-specific manner. Many top female-specific key drivers are immune-related genes such as *PTPRO*, *SH3BP2*, *TYROBP*, and *C8B* (Fig. 4c for liver, Fig. 4d for adipose). In contrast, male-specific key drivers show broader functional diversity including various aspects of metabolism (e.g., fatty acids—*FASN* and *CD36*, mitochondria—*CPT2* and *CHCHD6*), insulin signaling (e.g., *FASN*, *GYS1*), immune system (e.g., *INPP5D*, *FCER1G*, *NCKAP1L*), and cell growth and apoptosis (*ANXA2, CIDEC*) (Fig. 4e for liver, Fig. 4f for adipose). Notably, both the shared and sex-specific key driver subnetworks contain numerous known human GWAS genes such as *PNPLA3* and *TRIB1*, but the GWAS genes are less likely to be key drivers. The combined subnetworks of NAFLD processes that are female-specific or shared between sexes are given in Additional file 7 A and B for liver and adipose tissue, respectively, whereas the male-specific or shared networks were previously presented in [18].

### Potential regulation of the NAFLD networks by sex hormones

To explore the potential role of sex hormones in regulating the sexually dimorphic NAFLD genes and pathways identified, we tested the sex-specific liver and adipose tissue key driver subnetworks for enrichment of known target genes of estrogen receptors (ESR1, ESRRA, ESRRB, and ESRRG) and androgen receptor (AR) based on FANTOM5 transcription factor regulatory networks [44] from matching tissues. We found that female-specific liver and adipose NAFLD networks were strongly enriched with target genes of estrogen receptors and male-specific key driver subnetworks showed a strong enrichment for the target genes of AR in both tissues (Additional file 8). We also investigated whether individual key drivers in the sex-specific networks are targets of sex hormones or show interactions with sex hormones. Among the female-specific key drivers, *NCKAP1L* is a target of estrogen receptor ESRRA; *C8B* is a target of multiple estrogen receptors ESRRA, ESRRB, and ESRRG; and *SH3BP2* is a target of ESR1 [44]. Among the male-specific key drivers, *CHCHD6* is a target gene of the androgen receptor. These results suggest that sex hormones may regulate the sex-specific key drivers and pathways and partially explain the sexual dimorphism in NAFLD.

### Gonadectomy and ovariectomy experiments support the regulatory roles of sex hormones in NAFLD sexual dimorphism

To more directly test the role of sex hormones, we compared our NAFLD subnetworks with the transcriptome of both liver and adipose tissues from gonadectomized and ovariectomized mice. At FDR < 5%, we identified 2435 and 196 differentially expressed genes in liver tissue of males and females, respectively, and 1804 and 1491 differentially expressed genes in adipose tissue of males and females, respectively (Table 1). These results suggest a significant impact of testosterone on both male tissues and estrogen on the female adipose tissue. In contrast, female liver tissue was less affected by estrogen deficiency. We analyzed the enrichment of each differentially expressed gene list in our sex-specific NAFLD subnetworks (Fig. 4c–f) for the corresponding sex and tissue. Consistent with the numbers of differentially expressed genes from the gonadectomy/ovariectomy experiments, the female liver NAFLD subnetworks (Fig. 4c) had the smallest number of genes (86 compared to hundreds in the other networks). Only two genes in this subnetwork were found to be affected by estrogen deficiency in the female liver. In stark contrast, about half of the male liver NAFLD subnetwork genes (100 of 218 genes) were found to be affected by testosterone deficiency in male liver tissue. Similarly, significant overlaps between the adipose NAFLD subnetwork genes and the adipose genes affected by sex hormone deficiency were observed for both sexes. Besides, some of our top key driver genes were among the differentially expressed genes for the corresponding tissue and sex as listed in Table 1 (see also Fig. 4c–f). These results support that sex hormones are involved in the regulation of the male-specific NAFLD processes in both tissues but only adipose pathways in females.

**Table 1:** Overlap between sex-specific NAFLD subnetworks and DEGs affected by sex hormones

Tissue	Sex	Network size	DEG size	Overlap gene size (overlap KD size)	Overlap KD list	Fold change	*P* value
Liver	M	218	2435	100 (14)	*C8B*, *CYP7B1*, *SLC16A7*, *SLC16A5*, *CD36*, *MGST3*, *NCKAP1L*, *INPP5D*, *ANXA2*, *HCK*, *FCER1G*, *FGL2*, *CIDEC*, *TBC1D15*	6.13	8.28E−71
	F	86	196	2 (0)	–	3.86	8.68E−02
Adipose	M	261	1804	71 (8)	*FASN*, *AACS*, *ETFDH*, *GYS1*, *ECHS1*, *SH3D21*, *SLC2A3*, *PMEPA1*	4.39	9.34E−35
	F	206	1491	34 (5)	*MSLN*, *GPM6A*, *PTGIS*, *RSPO1*, *BNC1*	3.22	4.57E−13

One-sided Fisher’s exact test was used to calculate enrichment *P* values

## Discussion

To understand the sexual dimorphism observed in NAFLD, here, we compared the genetically perturbed mechanisms in NAFLD between sexes via integration of multi-omics data including GWAS, tissue-specific transcriptomic and eQTL data, and NAFLD phenotypic data from > 100 inbred and recombinant inbred mouse strains for females and males. This comprehensive data-driven analysis revealed both shared and sex-specific pathways, regulatory genes, and networks, thereby providing molecular insights into the sex differences in NAFLD. Furthermore, both our in silico FANTOM5 transcription factor analysis and in vivo hormone modulation experiments support a strong role of sex hormones in the regulation of sex-specific NAFLD pathways.

We conducted our analysis in a tissue and sex-specific manner, identifying and comparing causal NAFLD processes between sexes in the liver and adipose tissue since those are the most relevant and implicated tissues in NAFLD pathogenesis [48]. We observed numerous processes that are shared by both sexes, such as the cell cycle, apoptosis, and lipid metabolism in both tissues, fatty acid metabolism, oxidative phosphorylation, and growth factor receptor signaling in liver tissue, and branched-chain amino acid metabolism, adaptive immune system, and post-translational protein modification in adipose tissue (Additional file 4). Many of these pathways have been previously associated with NAFLD [48, 58, 59], confirming the reliability of our approach. The consistency of these shared terms between sexes highlights them as the core processes that can be targeted generally for NAFLD.

To facilitate the identification of potential targets for the above-shared pathways, we used a network modeling approach to define candidate regulator genes or key drivers. The predicted key drivers were found to orchestrate genes in the NAFLD processes shared by both sexes, forming highly connected subnetworks containing numerous previously known NAFLD genes (such as *AHSG*, *FDFT1*, *FASN*, *ACADVL*, *PNPLA3*, *GCKR*, *LYPLAL1*, and *LCP1*) (Fig. 4a, b). In liver tissue, many key drivers are involved in fatty acid and cholesterol metabolism, such as *ACOT2* and *DHCR7*. In adipose tissue, notable key drivers such as *BCKDHA*, *MCCC1*, and *ECHS1* are key enzymes involved in branched-chain amino acid catabolism. Branched-chain amino acids are known to be involved in numerous physiological activities related to nutrition, metabolism, and immune system processes and metabolic disorders such as insulin resistance and NAFLD. Impairments in branched-chain amino acid degradation deteriorate the TCA cycle, which may lead to mitochondrial dysfunction in NAFLD [60]. Identification of the key drivers that regulate the shared pathogenic processes between sexes may facilitate the prioritization of targets to treat the disease in the general population.

In addition to the shared pathways, we observed female-specific processes in the liver and adipose tissue (Additional file 4) and predicted their potential regulators. NCK-associated protein 1-like (*NCKAP1L*) and TYRO protein tyrosine kinase binding protein (*TYROBP*) were found to be the top key drivers of the innate immune system in the liver. The same key drivers have been previously found to regulate an inflammation network involved in a large number of diseases including diabetes, obesity, cardiovascular disease, and cancers [61]. Another top key driver regulating a female-specific process in the liver is complement C8 beta chain (*C8B*) in the complement pathway. It has not been previously associated with NAFLD; however, it is connected to three human NAFLD GWAS genes (*GCKR*, *LYPLAL1*, and *TM6SF2*) in our liver network, making it a strong novel candidate target for NAFLD. SH3 domain-binding protein 2 (*SH3BP2*), is a female-specific adipose key driver for the TCR signaling and lysosome module. *SH3BP2* has not been previously associated with NAFLD to the best of our knowledge, but it is a target of estrogen receptor 1 according to FANTOM5 adipose tissue gene regulatory network [44], which may support our sex and tissue-specific finding on *SH3BP2.*

Notably, lipid-related processes (e.g., phospholipid, lysophospholipid) are prominent in males (Additional file 4) [18] but not in females. Another male-specific process, Notch signaling, was previously found to be correlated with insulin resistance, hepatic steatosis, alanine aminotransferase, and NAFLD activity score in liver biopsies [62]. Our results support a causal role of this pathway in NAFLD development, particularly in males. We also observed male-specific adipose tissue pathways such as Wnt and insulin signaling, which agrees with the observations that insulin resistance is strongly correlated with NAFLD mainly in males and that males with NAFLD have higher glucose and lower adiponectin levels than females [11]. Differences in insulin sensitivity may partially explain sex differences in NAFLD. Male-specific top key drivers include *CHCHD6* and *CD36* in liver and *FASN* and *CPT2* in adipose tissue, which primarily regulate mitochondrial function and fatty acid metabolism. Coiled-coil-helix-coiled-coil-helix domain containing 6 (*CHCHD6*) was experimentally validated in our recent study as a novel key regulator of NAFLD by impairing mitochondrial functions in the liver [18]. These male-specific pathways and regulators along with the female-specific ones discussed earlier can facilitate future efforts to investigate the sex-specific mechanisms in NAFLD and may serve as potential therapeutic targets for sex-dependent treatments.

It is noted that estrogen deficiency in mice fed a high-fat diet leads to accelerated NAFLD progression [13], and after menopause, the rate and severity of NAFLD increases in females [10, 12]. Testosterone has also been associated with a protective role in NAFLD in males [46, 63, 64]. Since both male and female sex hormones might have a role in slowing the NAFLD progression, we also investigated the potential effects of the sex hormones on the sexual dimorphism in NAFLD using both an in silico transcription factor analysis and in vivo gonadectomy/ovariectomy experiments. Both analyses support significant overlaps between the sex-specific liver and adipose tissue NAFLD networks and sex hormone target genes. The significant overlaps also include our top key driver genes for the corresponding tissue and sex (Table 1, Fig. 4c–f). These results support a role of sex hormones in the regulation of the sex-specific NAFLD networks and pathways. However, hormones only partially explain the sex-specific networks. In the case of females, ovariectomy had limited effects on liver gene expression, suggesting minor contribution of estrogen deficiency to the female-specific liver networks. Since the ovariectomy experiment was carried out in adulthood, it is possible that developmental estrogen exposure plays a stronger role in shaping female liver network. It is also plausible that sex chromosomes play an important role, particularly in the female liver. Further investigations are required to explore the role of sex chromosomes in regulating sexually dimorphic networks for NAFLD.

A major strength and novelty of our study is the utilization of a multi-omics integrative approach to identify genetically causal or perturbed sex- and tissue-specific processes and key regulatory genes in NAFLD based on genetics, functional genomics, and gene regulatory networks. The integration of multidimensional datasets enabled us to derive one of the most comprehensive views of the genes and pathways that are likely perturbed by genetic risks of NAFLD in both males and females, thereby significantly enhancing our understanding of sexual dimorphisms in NAFLD. Compared to a previous study exploring sex differences in the same animal cohort, which focused on a few genome-wide significant loci and pair-wise correlations between phenotypes and individual genes [46], our study uniquely leveraged the full spectrum of molecular associations across multi-omics dimensions and benefited from network representations of regulatory relationships. In addition to confirming mitochondrial function, oxidative phosphorylation, and transmembrane transport as important NAFLD processes [46], we identified numerous sex-specific genes and pathways that were missed in the previous study.

A principle limitation of our study is that our analytical framework only considers the effect sizes but not the directionality of NAFLD GWAS associations and gene expression changes. As such, it is difficult to infer if the sex-specific pathways are protective or pathogenic for NAFLD. For example, it is possible that some of the sex-specific pathways reduce instead of promote disease risks. Building on our recent successes in experimentally validating predictions from the integrative genomics approach described here, future studies that perturb individual sex-specific pathways and key drivers identified here will help confirm the causality of the predicted genes and pathways and determine whether they should be activated or inhibited to ameliorate NAFLD.

Our sex-specific findings help understand the long-observed sexual dimorphism in NAFLD and provide insights into the potential differential pathogenic pathways between males and females, which can guide future development of sex-specific therapeutics in translational studies. Although our study is carried out in mice, there is strong evidence for replication of our findings in human studies. For instance, results from our previous male-focused study [18] are consistent with an independent human study demonstrating the functional associations of *FASN*, *PKLR*, and *THRSP* with NAFLD [65]. Additionally, our NAFLD gene subnetworks (Fig. 4) contained human NAFLD GWAS genes such as *PNPLA3*, *LCP1*, and *TMSF2*. Furthermore, our recent study examining liver fibrosis identified significant overlaps in the genes and pathways between mouse and human [66]. These observations support the translatability of our findings from mouse studies to human NAFLD or NASH pathogenesis.

## Conclusions

The National Institute of Health and the Food and Drug Administration have prioritized personalized medicine as a key to developing effective therapies [67]. Sex is one of the important factors to consider when developing personalized medicine as it can affect drug dose level, adverse reactions, and responses. There are numerous reported sex differences in liver disease drugs with regard to the rate of response and adverse reactions, but few of these sex differences are understood [12]. Our multi-omics integrative approach combining genetic, gene expression, functional genomics, and network modeling delineated genes, pathways, and tissue-specific networks that contribute to the sexual dimorphism of NAFLD. Our findings support the existence of core pathogenic processes that can be targeted in both sexes and pinpoint sex-specific mechanisms to guide differential therapeutic options for males and females to more effectively mitigate NAFLD in a personalized manner.

## Additional files

Additional file 1: Z-summary scores for the module preservation of the female and male coexpression modules for both MEGENA and WGCNA methods in each tissue. Preservation of the female MEGENA modules in male MEGENA modules for (A) Liver and (B) Adipose tissues. Preservation of female WGCNA modules in male WGCNA modules for (C) Liver and (D) Adipose tissues. Preservation of the male MEGENA modules in female MEGENA modules for (E) Liver and (F) Adipose tissues. Preservation of male WGCNA modules in female WGCNA modules for (G) Liver and (H) Adipose tissues. (I) Mutual preservation ratios of female and male coexpression modules for both methods and both tissues. (PDF 345 kb)

Additional file 2: GO annotation of the hepatic triglyceride-related modules (based on eigen gene and trait correlation) (XLSX 32 kb)

Additional file 3: Raw MSEA results. Left Panel shows the liver tissue results, while right panel shows the adipose tissue results. (XLSX 14 kb)

Additional file 4: MSEA and KDA results of the sex-specific, tissue-specific, and shared supersets between sexes in each tissue. A superset is a gene set merging a group of highly overlapping pathways or coexperssion modules to reduce redundancy. Coexpression modules are labeled with IDs from WGCNA or MEGENA network and annotated with GO terms. (XLSX 14 kb)

Additional file 5: KDA results. Left Panel shows the liver tissue results, while right panel shows the adipose tissue results. (XLSX 71 kb)

Additional file 6: Previously annotated NAFLD-associated genes from the literature. Genes that were identified as a key driver in our analysis are highlighted in particular colors as denoted below. (XLSX 18 kb)

Additional file 7: Combined subnetworks of NAFLD processes that are shared between sexes or female-specific. (A) Liver Bayesian subnetwork comprised of NAFLD supersets that are perturbed in liver tissue of females (as a combination of female-specific processes or shared processes between sexes) and the top key drivers of each superset. (B) Adipose Bayesian subnetwork comprised of NAFLD supersets that are perturbed in adipose tissue of females (as a combination of female-specific processes or shared processes between sexes) and the top key drivers of each superset. (PDF 4683 kb)

Additional file 8: Enrichment of the key driver subnetworks for the target genes of the sex hormone receptors. Enrichment is evaluated in a tissue-specific manner using the tissue-specific FANTOM5 gene regulatory networks. (XLSX 9 kb)

## Abbreviations

eQTL: Expression quantitative trait loci
GWAS: Genome-wide association studies
HMDP: Hybrid mouse diversity panel
KDA: Key Driver Analysis
MSEA: Marker Set Enrichment Analysis
NAFLD: Non-alcoholic fatty liver disease
NASH: Nonalcoholic steatohepatitis
