# Supplementary material for: Tissue-specific pathways and networks underlying sexual dimorphism in non-alcoholic fatty liver disease
Source: Biol Sex Differ. 2018 Oct 22;9:46. doi: 10.1186/s13293-018-0205-7 (PMC6196429; doi:10.1186/s13293-018-0205-7)
Supplement: Supplementary file 1 — Z-summary scores for the module preservation of the female and male coexpression modules for both MEGENA and WGCNA methods in each tissue. Preservation of the female MEGENA modules in male MEGENA modules for (A) Liver and (B) Adipose tissues. Preservation of female WGCNA modules in male WGCNA modules for (C) Liver and (D) Adipose tissues. Preservation of the male MEGENA modules in female MEGENA modules for (E) Liver and (F) Adipose tissues. Preservation of male WGCNA modules in female WGCNA modules for (G) Liver and (H) Adipose tissues. (I) Mutual preservation ratios of female and male coexpression modules for both methods and both tissues. (PDF 345 kb) [file 13293_2018_205_MOESM1_ESM.pdf]

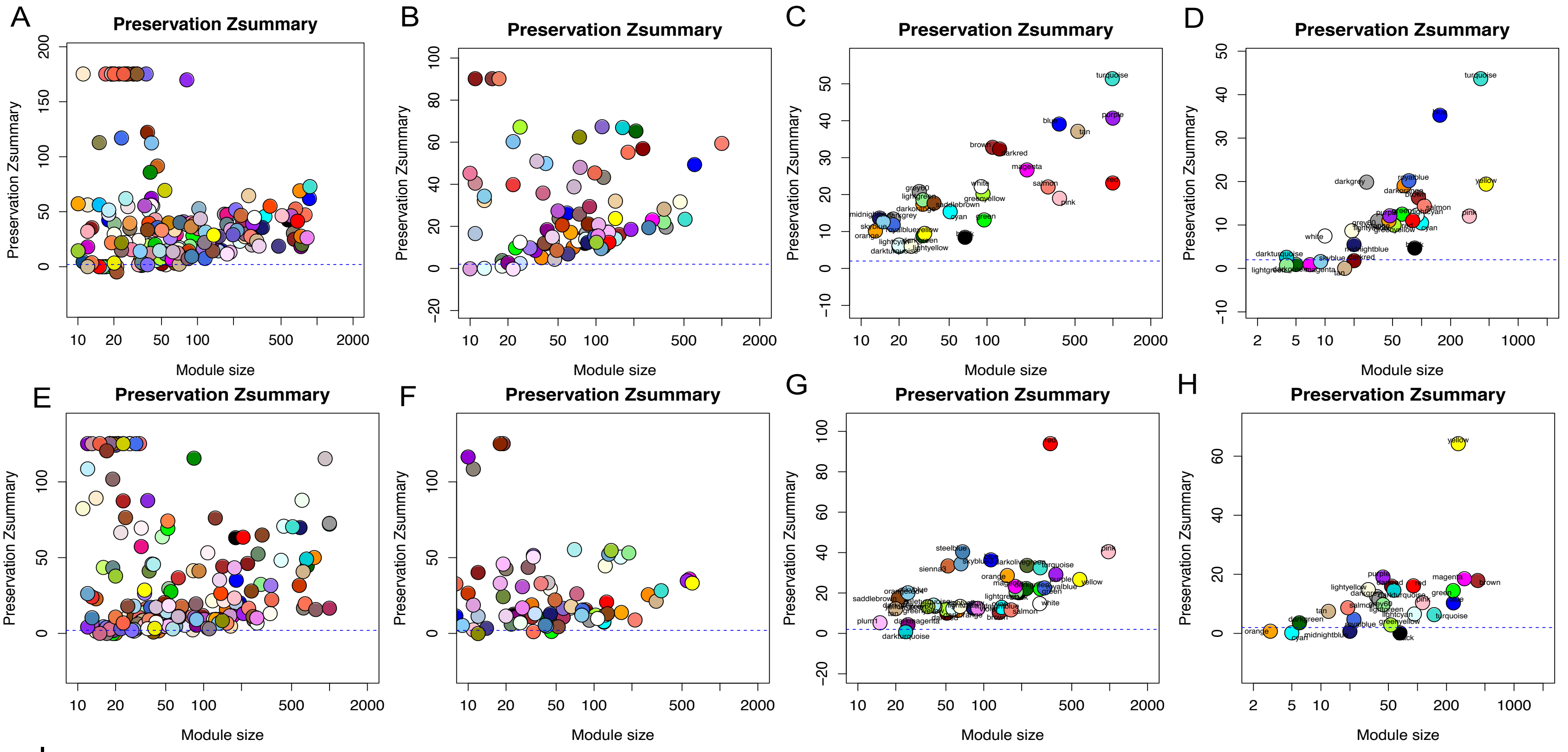

I

Tissue	Network type	Femalemodules preservation in maleexpression data	Malemodules preservation in Femaleexpression data	
Liver	WGCNA	0modules non-conserved (0%)<br>30modules conserved (100%)	1module non-conserved (3%)<br>40modules conserved (97%)	Avg. in liver across sexes:
	MEGENA	15modules non-conserved (7%)<br>198modules conserved (93%)	12modules non-conserved (6%)<br>192modules conserved (94%)	
	Avg. in each sex	Average in femaleliver:<br>4%non-conserved<br>96%conserved	Average in maleliver:<br>5%non-conserved<br>95%conserved	5% non-conserved<br>95% conserved
Adipose	WGCNA	6modules non-conserved (20%)<br>24modules conserved (80%)	4modules non-conserved (15%)<br>26modules conserved (85%)	Avg. in adipose tissue across sexes:
	MEGENA	4 modules non-conserved (5%)<br>81modules conserved (95%)	3modules non-conserved (4%)<br>79modules conserved (96%)	
	Avg. in each sex	Average in femaleadipose:<br>13%non-conserved<br>87% conserved	Average in maleadipose:<br>10%non-conserved<br>90%conserved	12% non-conserved<br>88% conserved

Additional File 1: Z-summary scores for the module preservation of the female and male coexpression modules for both MEGENA and WGCNA methods in each tissue. Preservation of the female MEGENA modules in male MEGENA modules for (A) Liver and (B) Adipose tissues. Preservation of female WGCNA modules in male WGCNA modules for (C) Liver and (D) Adipose tissues. Preservation of the male MEGENA modules in female MEGENA modules for (E) Liver and (F) Adipose tissues. Preservation of male WGCNA modules in female WGCNA modules for (G) Liver and (H) Adipose tissues. A Z-summary score <2 (dashed blue line) shows no preservation, whereas a Z-summary score >2 shows evidence for the preservation. (I) Mutual preservation ratios of female and male coexpression modules for both methods and both tissues.
