# Supplementary material for: Tissue-specific pathways and networks underlying sexual dimorphism in non-alcoholic fatty liver disease
Source: Biol Sex Differ. 2018 Oct 22;9:46. doi: 10.1186/s13293-018-0205-7 (PMC6196429; doi:10.1186/s13293-018-0205-7)
Supplement: Supplementary file 7 — Combined subnetworks of NAFLD processes that are shared between sexes or female-specific. (A) Liver Bayesian subnetwork comprised of NAFLD supersets that are perturbed in liver tissue of females (as a combination of female-specific processes or shared processes between sexes) and the top key drivers of each superset. (B) Adipose Bayesian subnetwork comprised of NAFLD supersets that are perturbed in adipose tissue of females (as a combination of female-specific processes or shared processes between sexes) and the top key drivers of each superset. (PDF 4683 kb) [file 13293_2018_205_MOESM7_ESM.pdf]

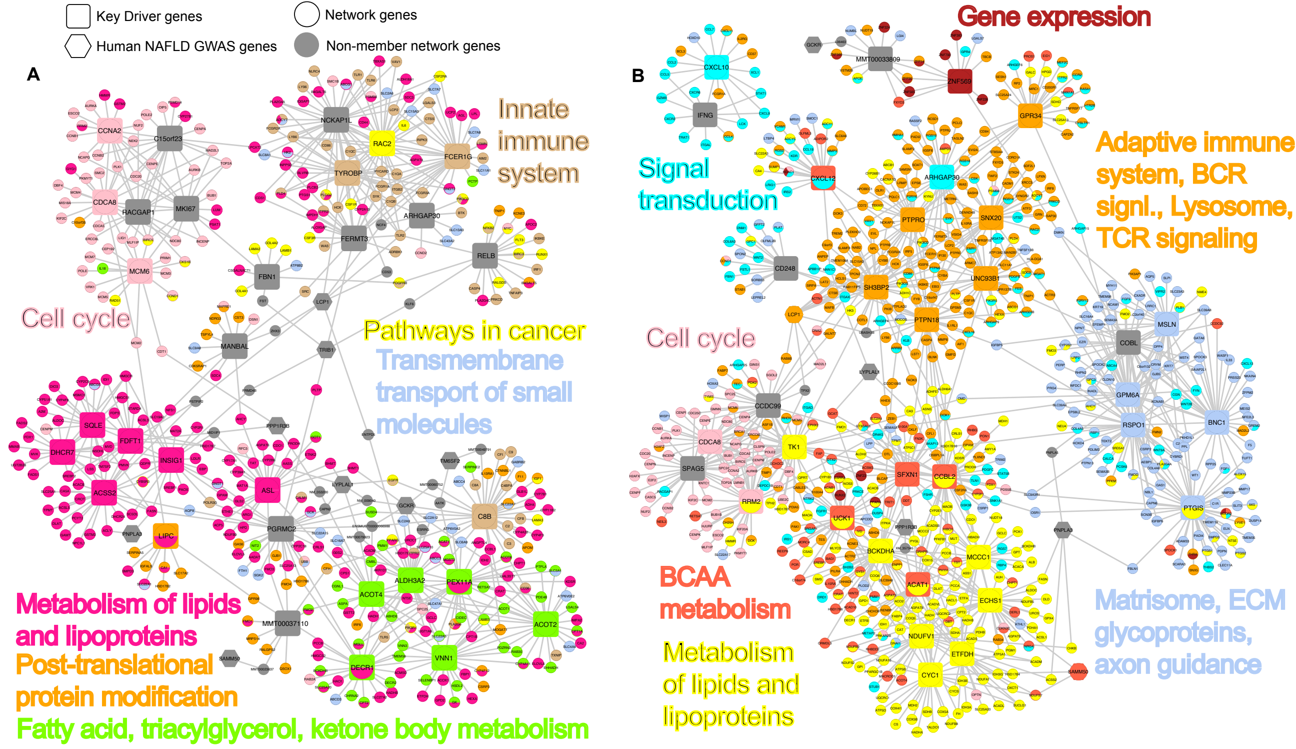

Additional File 7. Combined subnetworks of NAFLD processes that are shared between sexes or female-specific. (A) Liver Bayesian subnetwork comprised of NAFLD supersets that are perturbed in liver tissue of females (as a combination of female-specific processes or shared processes between sexes) and the top key drivers of each superset. **(B)** Adipose Bayesian subnetwork comprised of NAFLD supersets that are perturbed in adipose tissue of females (as a combination of female-specific processes or shared processes between sexes) and the top key drivers of each superset.
